# The association between Femoral Tilt and impingement-free range-of-motion in total hip arthroplasty

**DOI:** 10.1186/1471-2474-13-65

**Published:** 2012-05-04

**Authors:** Tobias Renkawitz, Martin Haimerl, Lars Dohmen, Sabine Gneiting, Philipp Lechler, Michael Woerner, Hans-Robert Springorum, Markus Weber, Patrick Sussmann, Ernst Sendtner, Joachim Grifka

**Affiliations:** 1 Department of Orthopaedic Surgery, Regensburg University Medical Center, Regensburg, Germany; 2 Brainlab AG, R&D Surgery, Feldkirchen, Germany

## Abstract

**Background:**

There is a complex interaction among acetabular component position and antetorsion of the femoral stem in determining the maximum, impingement-free prosthetic range-of-motion (ROM) in total hip arthroplasty (THA). By insertion into the femoral canal, stems of any geometry follow the natural anterior bow of the proximal femur, creating a sagittal Femoral Tilt (FT). We sought to study the incidence of FT as measured on postoperative computed tomography scans and its influence on impingement-free ROM in THA.

**Methods:**

The incidence of the postoperative FT was evaluated on 40 computed tomography scans after cementless THA. With the help of a three-dimensional computer model of the hip, we then systematically analyzed the effects of FT on femoral antetorsion and its influence on calculations for a ROM maximized and impingement-free compliant stem/cup orientation.

**Results:**

The mean postoperative FT on CT scans was 5.7° ± 1.8°. In all tests, FT significantly influenced the antetorsion values. Re-calculating the compliant component positions according to the concept of combined anteversion with and without the influence of FT revealed that the zone of compliance could differ by more than 200%. For a 7° change in FT, the impingement-free cup position differed by 4° for inclination when the same antetorsion was used.

**Conclusions:**

A range-of-motion optimized cup position in THA cannot be calculated based on antetorsion values alone. The FT has a significant impact on recommended cup positions within the concept of “femur first” or “combined anteversion”. Ignoring FT may pose an increased risk of impingement as well as dislocation.

## Background

**Figure 1:**
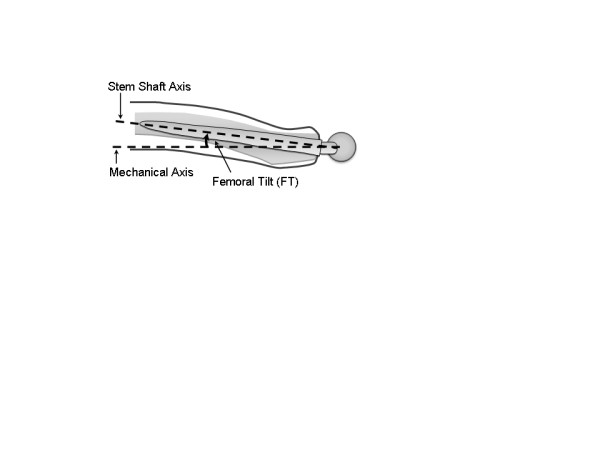
** Calculation of Femoral Tilt.** FT is calculated as the deviation between mechanical axis and shaft axis in a sagittal projection.

One of the great intraoperative challenges in total hip arthroplasty (THA) is to find an optimized combination of hip biomechanics, tribology, and post-operative functionality. Component malpositioning and soft tissue imbalance influence the prevalence of dislocation and impingement between the bone and/or the prosthesis [1-4]. Multifold models have been developed to determine the optimal combination of cup inclination, cup anteversion, and stem antetorsion for maximizing range-of-motion (ROM) and minimizing the risk for impingement. In this context, different authors have proposed to start with the preparation of the femur (“femur first”) and adjust the position of the acetabular cup in accordance with the femoral stem rotation [5-8]. Previous studies have shown that, with common cementless hip stems, the surgeon has virtually little control of the antetorsion of the femoral component [7]. Depending on the anatomical shape of the femur, the broaches and the implant virtually “find their way” to a position, where the stem conforms best to the rigid shape of the native proximal femur canal. The rotational movement results in a wide variability of stem antetorsion from 15° of retroversion to 45° of anteversion as measured on the postoperative CT scans [9,10]. In the sagittal direction, the stem follows the natural anterior bow of the proximal femur during insertion into the medullary canal, which creates a deviation between the femoral shaft and the mechanical axis in a sagittal projection. This is best described as “Femoral Tilt” (FT) (Figure 1). So far, the influence of FT on stem antetorsion, impingement, and ROM in THA has not yet been analyzed to it’s full extend.

The purpose of the current study was therefore threefold:

1. To study the postoperative incidence of FT for cementless femoral stems on computed tomography scans.

2. Systematically analyze the effects of FT on femoral antetorsion by means of a three-dimensional computer model.

3. Re-calculate the zones of impingement-free compliant stem/cup orientation with and without the effect of FT.

## Methods

### Study population

For studying the incidence of FT after cementless THA, 40 postoperative computed tomography (CT) scans were analyzed by a single investigator (LP). This study was conducted after authorization by the Institutional Ethical Board (No. 06/100) and the Federal Office for Radiation Protection (Z5-22462/2-2007-008) and informed consent was obtained from all patients. Average patient age was 69 (±4.8) years and average body mass index (BMI) 26.4 (±3.7) kg/m^2^. Exclusion criteria were arthritis secondary to hip dysplasia, post-traumatic deformities of the pelvis, and - because a post-operative pelvic CT scan was required - age below 50 years at the time of surgery. All operations were done with the patient in lateral position through a modified Smith-Petersen (Micro-Hip®) approach [11] by two surgeons (TR, ES). Press-fit components and cement-free hydroxyapatite-coated stems (Pinnacle cup, Corail stem, DePuy, Warsaw, IN, USA) were used in all cases. The CT data sets (pelvis and femoral condyles) included 16 male and 24 female, 17 left and 23 right hips. None of the patients had a THA on both sides.

### Alignment of stem implant and planning of landmarks

**Figure 2:**
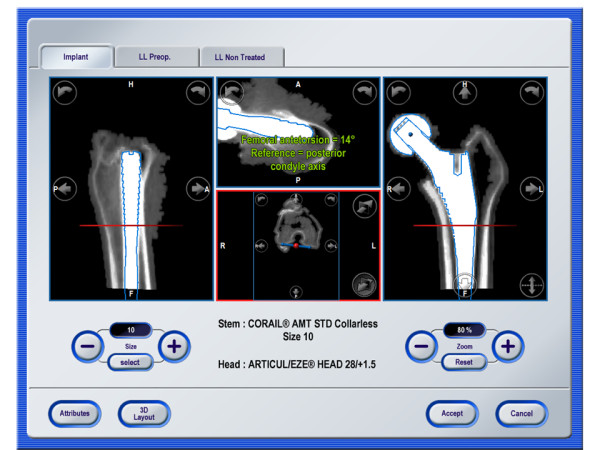
** Alignment of stem implant and planning of landmarks.** The geometric CAD model of the implant is superimposed onto the CT data set and antetorsion is measured as the angle between the posterior condyle axis landmarks and the implant neck axes in an axial projection.

A three-dimensional (3D) CT analysis software (Hip CT 3.5.2, Brainlab AG, Feldkirchen, Germany) was used to evaluate the incidence of FT. The software included an implant database with computer-aided design (CAD) 3D models of the implant geometries provided by the implant manufacturer. The models of the actual implants were superimposed onto the image data to determine their exact position, i.e. the implants were manually aligned until the geometric models fitted optimally to the actual implants (Figure 2). According to the implant data stored in the database, the orientation of the neck and shaft axis of the stem was then assessed and analyzed. In situ orientation was determined by anatomical landmarks. The center of the implant’s head was determined by manually placing a sphere around the head in the image data. The transepicondylar axis was constructed between the most prominent aspects of the femoral condyles, visible in the horizontal plane. The mechanical axis of the femur was determined by the connecting line between the center of the femoral head and the midpoint of the transepicondylar axis. The posterior condyles were planned as the most posterior points of the femoral condyles in the CT data sets. This axis was used to complete the coordinate system of the femur, i.e. the coronal plane was defined as the plane spanned by the mechanical and the direction of the posterior condyle axis. Femoral Tilt (FT) was then calculated as the deviation between the mechanical axis and shaft axis of the stem in a sagittal projection.

### Creation of a geometric model to represent the orientation of the stem implant

**Figure 3:**
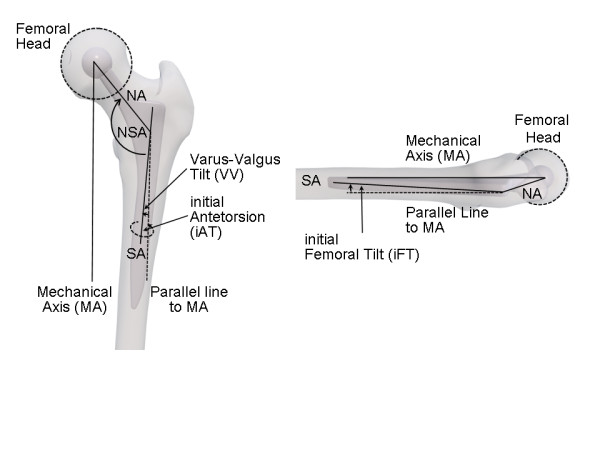
** Geometric model to represent the orientation of the stem implant.** Left: coronal view, right: sagittal view. First, a given neck-shaft angle (NSA) angle was applied. Then, the orientation of the stem implant was achieved by the following rotations: rotation around the cranial-caudal axis by the initial antetorsion (iAT) angle, rotation around the medial-lateral direction by the initial femoral tilt (iFT) angle, and rotation around the anterior-posterior axis by the varus-valgus (VV) angle. The cranial-caudal direction was defined by the mechanical axis and the coronal plane by the plane spanned by the mechanical axis (MA) and the direction of the posterior condyle axis. Further abbrevations: neck axis (NA), proximal shaft / stem axis (SA).

For the second part of the analysis, a three-dimensional (3D) computer model of the hip was used to systematically analyze changes in the femoral anatomy and its effects on femoral antetorsion (AT). In our model, we defined FT as the angle, which directly reflects the deviation between the proximal femoral shaft and the mechanical axis in a sagittal projection. Figure 3 shows the construction of the model including an initial FT (iFT) and initial AT (iAT) reflecting angles derived from a rotational representation of the neck axis alignment. According to Yoshioka [12], AT was defined as the deviation between the neck axis and the posterior condyle axis when projected to a plane orthogonal to the mechanical axis. As a first step, the effect of changes in iFT on the resulting AT angle was analyzed (Additional file 1). For this purpose, iFT values were stepwise increased from 2.1° to 9.3° for three values of iAT (0°, 15°, 30°). Following our analysis of FT on the postoperative CT scans, an iFT angle of 5.7° was considered to be an average value, whereas 2.1° and 9.3° represented lower and upper margins. VV was fixed to 4.5° in these experiments. Further on, differences between the initial (iFT) and true FT values were analyzed in the range 2.1° to 9.3° iFT and 0° to 10° VV. Respectively the zone of impingement-free, compliant cup positions was determined for stem positions with varying iFT according to the approach as published by Widmer [8]. This so called “zone-of-compliance” contains a combined, stem/cup orientation to position both components in a way that the normal range-of-motion (ROM) is contained within an impingement-free prosthetic ROM, aiming for at least 130° flexion, 40° extension, 50° abduction, 50° adduction, 40° external, and 80° internal prosthetic rotation. Additionally, an impingement-free prosthetic ROM for int./ext. rotation at 90° of flexion was taken into account. The limits were set to 45° for internal and 55° for external rotation. This intended prosthetic ROM is larger than the movements in commonplace maneuvers known to increase the risk for dislocation in THA [2,3]. As proposed by Widmer et al., a restriction of the inclination (≤ 50°) was used as an additional constraint. ROM was calculated by an algorithm which determines collisions between the femoral and cup implant. This algorithm was implemented into a prototype software (Hip Storage Viewer, Brainlab AG, Feldkirchen, Germany). The geometry of the implants was specified by 3D CAD files (geometric 3D models) provided by the implant manufacturer. Conventional cementless implants [Pinnacle cup, standard Corail stem (NSA 135°), neutral polyethylen liner, short 32 mm head; DePuy, Warsaw, IN, USA] were used. ROM was calculated based on a neutral position of the leg by aligning the femoral and the pelvis coordinate system. The analysis did not depend on a particular coordinate system of the pelvis since only the orientation of the implants was considered.

### Comparison between femoral tilt and zones-of-compliance

As a last step, resulting zones-of-compliance were compared for two variations in iFT (2.1°, 9.3°). The same (effective) AT of 15° was used for this analysis, i.e. the antetorsion was adapted according to the variations in iFT. This allowed assessing differences in the zones-of-compliance when equal (effective) antetorsion values but different iFT values are given. In analogy to Widmer et al., the optimum cup position was determined as the point with the lowest inclination within the zone-of-compliance where a safety zone of approximately 1° was respected. All cup orientation angles were calculated in terms of the radiographic definition according to Murray [13].

### Statistical analysis

Statistical analyses were performed using Microsoft Excel (Microsoft Inc, Redmond, WA, USA). Mean values, standard deviations, ranges, and confidence intervals with 95% confidence level were calculated. Statistical differences between the group of male and female patients were analyzed by a Student’s two sample t-tests assuming equal variances (significance level: 5%).

## Results

Table 1 gives an overview about the incidence of FT after cementless THA on 40 postoperative CT scans. There was no significant difference between male and female patients (p < 0.05). When iFT was changed, the effect on antetorsion, i.e. the difference between AT and iAT, was found to be in the range between −10.0° and 0.6°. The incremental changes were found to be between −1.15° and −0.95°, i.e. an increment of 1° in iAT results in a decrease of approximately 1° in AT. The differences between iFT and FT were below 0.3° in the considered range 2.1°–9.3° iFT and 0°–10° VV. Thus, both definitions can be considered as approximately equivalent.

**Table 1:** Incidence of Femoral Tilt as measured on 40 postoperative CT scans after cementless total hip arthroplasty

	**Mean**	**StDev**	**95% Confidence interval**	**Range**
Female	5.7°	1.9°	2.0°–9.4°	1.7°–10.2°
Male	5.8°	1.8°	2.3°–9.3°	2.1°–8.0°
Total	5.7°	1.8°	2.1°–9.3°	1.7°–10.2°

**Figure 4:**
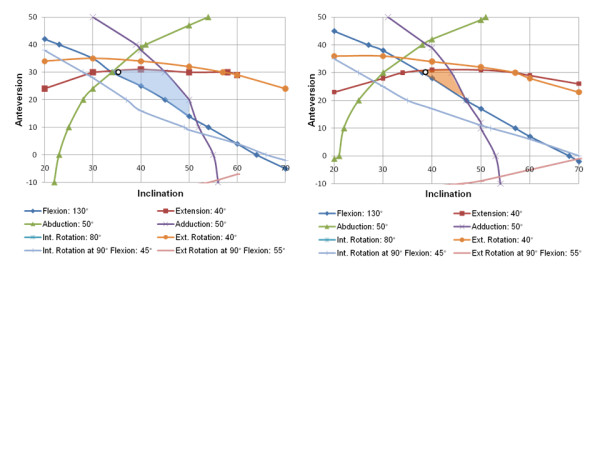
** Zones of impingement-free compliant stem/cup orientation under the influence of (initial) Femoral Tilt.** Left side: iFT = 2.1°, right side: iFT = 9.3°. In both cases the effective antetorsion (AT) is set to 15° and NSA = 135°, VV = 4.5°. The zone-of-compliance is more than 200% smaller for iFT = 9.3°. The optimum cup position according to Widmer (marked by a circle) changes from 35° radiographic inclination / 30° anteversion to 39° inclination / 30° anteversion.

Additionally, changes of FT had an impact on the compliant cup positions according to Widmer et al.. The area for the impingement-free zone-of-compliance decreased significantly by more than 200% when iFT was increased by 7.2° from 2.1° to 9.3° (Figure 4). At the same time, the optimum cup position according to the combined anteversion approach changed from 35° radiographic inclination/30° anteversion to 39°/30° when the (effective) stem antetorsion was fixed.

## Discussion

Our CT based analysis of post-operative Femoral Tilt (FT) in cementless femoral stems revealed a considerable variability and maximum FT values up to 10.2°. We found no significant difference between male and female patients. Although our analysis in 40 patients is limited by numbers, it confirms that variations of the FT between 1.7° and 10.2° occur in clinical practice.

For analysing the effects of FT on femoral antetorsion and an impingement-free compliant stem/cup position, we used a three-dimensional hip joint model including CAD files of actual implants with specific geometries. Previous studies used generic implant models represented by spheres and cones. Within our computer model, we compared initial (iAT) versus effective antetorsion (AT) values. From a biomechanical perspective, the definition of iAT is based on a rotational approach to determine the implant orientation, whereas the definition of AT is a measurement which uses projections to an axial plane. Thus, basically a rotation-based definition of stem orientation (iAT) was compared with projection measurements (AT). From a clinical perspective, both definitions are useful. iAT reflects a direct rotation around the shaft axis of the stem. Thus, it corresponds to the rotational alignment of the stem in the proximal part of the femur, which e.g. can be modified by using modular implants. Instead, AT refers to the overall rotational alignment around a cranial-caudal axis. Our results show considerable alteration of femoral antetorsion with variation of FT, even when the same rotational alignment at the proximal part of the femur (iAT) is applied. For a neck-shaft angle of 135° this effect is almost 1-to-1, i.e. an increment of 1° in the FT angle induces a decrement of 1° in antetorsion (Additional file 1).

According to the results by Widmer at al., cup inclination, cup anteversion, and stem antetorsion determine an optimized, impingement-free ROM and are highly interdependent. Widmer determined a linear relationship between cup anteversion and stem antetorsion which has been summarized within the so-called “Widmer formula”: Cup anteversion + 0.7 x Stem antetorsion = 37.3 [5,8]. With the help of a 3D computer model of the hip, we were able to analyze these dependencies in terms of complementary component orientations with mating of the femoral head in the cup without impingement of the two throughout all body positions under the influence of FT. Our results clearly demonstrate that the size of this so-called “zone-of-compliance” can differ by more than 200% when clinical FT values are applied (Figure 4). As an example, the optimum cup position according to Widmer changes from 35° radiographic inclination to 39° inclination when a FT is increased by 7° and the same (effective) antetorsion is used. These findings are very important clinically, particularly for surgeons following the concept of “combined anteversion” or “femur first” in THA. A ROM-optimized cup position cannot be calculated based on antetorsion values only. Thus, if the surgeon were to position the cup in relation to the femoral stem antetorsion, the influence of FT has to be considered as well.

Apart from our previous work on this topic [14], there is only one study so far that has addressed the issue of sagittal femoral stem alignment [15]. This analysis with another cementless stem type was based on a different coordinate system, i.e. proximal femoral axis instead of mechanical axis which was used as a reference for neutral alignment of the leg in our study. In this study by Mueller, sagittal tilt was calculated as the deviation between the shaft axis of the proximal femur and the stem. The anterior bow of the femur, i.e. deviation between the proximal or stem shaft axis and mechanical axis of the femur, was neglected and only a comparison between the pre- and postoperative situation was performed. Additionally, antetorsion was defined according to a connecting line between the center of the femoral head and the proximal shaft axis in this study. Thus, these calculations by Mueller did not directly represent the orientation of the stem neck axis. Based on this definition, the effect of sagittal tilt on antetorsion did not only depend on the sagittal tilt of the stem but also on the rotation point, which was used for the comparison between the neutral reference position and the final orientation of the stem. In particular, the translational difference of the point at the top of the stem shaft axis then influences the antetorsion calculation. Such translations may be relevant for addressing bone-to-bone impingements since the position of the stem in the femoral canal may influence this. The definition of antetorsion in our study was directly based on the orientation of the stem neck axis, because the analysis was directed to the determination of ROM according to implant-to-implant impingement. Because of these differences, the effect of sagittal tilt on antetorsion was approximately 2-to-1 to 3-to-1 (for 131° neck-shaft angle) according to Mueller [15], i.e. 1° change in sagittal tilt changes the (effective) antetorsion by 2°–3°, whereas the relationship was approximately 1-to-1 (for 135° neck-shaft angle) and a bit lower (for 125° neck-shaft angle) in our study. Therefore, the results of our analysis and study by Mueller cannot be directly compared.

Our study has certain limitations. First, we manually superposed the implant models onto the CT images instead of directly defining the axes. Based on our experience, the alignment of the implants was more reproducible than the direct axis determinations as the implants can be aligned very clearly with the implants. Usually, the variation of implant alignment between different observers was in the order ≤1°. However, this was not evaluated in detail. Second, we considered only prosthetic impingement in a specific ROM. We did not assess functionality and clinical symptoms of impingement in our group of patients. Third, in addition to the influence of femoral tilt and stem antetorsion on post-operative ROM, stem tilting in the frontal plane (varus/valgus angle) influences the relationship between the shaft axis and the femoral coordinate system which defines the reference for assessing ROM [17]. Last, our radiological and biomathematical analysis was conducted for only one type of non-modular cementless stem.

## Conclusions

In summary, we have shown that there is a significant association between FT and an impingement-free ROM in THA. Additional parameters, such as neck-shaft angle, head-neck ratio, the design of the acetabular opening, and modular stems are additional parameters which influence impingement and ROM [16-18]. Therefore, our results can be used as an input for a next generation of computer-assisted navigation systems that couple FT and an individual three-dimensional impingement analysis to achieve patient specific, ROM optimized component orientation within the concept of femur first for THA [19].

## Competing interest

Dr. Renkawitz, Dr. Woerner, Dr. Lechler, Dr. Springorum, Dr. Sendtner and Prof. Grifka are affiliated only with the Department of Orthopaedic Surgery, Regensburg University Medical Center, Germany. Dr. Sussmann is also affiliated with the Schulthess Clinic, Switzerland. Dr. Haimerl, Mr. Dohmen, and Ms. Gneiting are affiliated only with Brainlab AG, Feldkirchen, Germany. This research project was supported by a grant by the German Federal Ministry of Education and Research (BMBF), grant number 01EZ 0915.

## Authors’ contributions

TR and MH: Conception and design of the study, acquisition of data, clinical and 3D analysis and interpretation of data, drafting the article, and final approval of the version to be submitted. LD: Implementation of algorithms for 3D-analysis and interpretation of data, revising the article critically for important intellectual content, and final approval of the version to be submitted. SG, PL: Acquisition of data, 3D-analysis and interpretation of data, revising the article critically for important intellectual content, and final approval of the version to be submitted. MW, H-RS, PS, ES, JG: Conception and design of the study and acquisition of data, clinical interpretation of data, drafting the article, and final approval of the version to be submitted. All authors read and approved the final manuscript.

## Pre-publication history

The pre-publication history for this paper can be accessed here:

http://www.biomedcentral.com/1471-2474/13/65/prepub

## Supplementary Material

Additional file 1 Dynamic model of the Femoral Tilt. FT is continuously increased from 0° to 10°. The resulting change of antetorsion is represented by the red line. Click here for file
